# 4-Nitro­phenol–piperazine (2/1)

**DOI:** 10.1107/S1600536813015328

**Published:** 2013-06-08

**Authors:** Perumal Nagapandiselvi, Srinivasan Muralidharan, Thothadri Srinivasan, Rengaswamy Goplalakrishnan, Devadasan Velmurugan

**Affiliations:** a Department of Physics, Anna University, Chennai 600 025, India; b Centre of Advanced Study in Crystallography and Biophysics, University of Madras, Guindy Campus, Chennai 600 025, India

## Abstract

In the title adduct, C_6_H_5_NO_3_·0.5C_4_H_10_N_2_, the piperazine ring possesses inversion symmetry and has a *chair* conformation. Its mean plane makes a dihedral angle of 65.45 (7)° with the 4-nitro­phenol ring. In the crystal, the piperazine ring is linked to two 4-nitro­phenol mol­ecules *via* O—H⋯N hydrogen bonds. The mol­ecules are also linked *via* bifurcated N—H⋯(O,O) hydrogen bonds involving the NO_2_ O atoms, forming a two-dimensional network lying parallel to (102). The networks are linked *via* C—H⋯O hydrogen bonds, forming a three-dimensional structure.

## Related literature

For the biological properties of piperazine compounds, see: Foroumadi *et al.* (2007 ▶); Upadhayaya *et al.* (2004 ▶); Chen *et al.* (2006 ▶); Cunico *et al.* (2009 ▶); Smits *et al.* (2008 ▶); Becker *et al.* (2006 ▶).
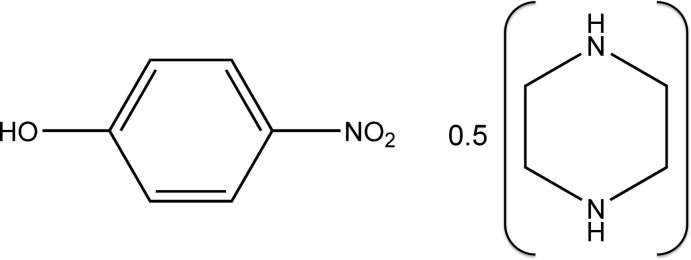


## Experimental

### 

#### Crystal data

C_6_H_5_NO_3_·0.5C_4_H_10_N_2_
*M* *_r_* = 182.18
Monoclinic, 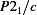

*a* = 6.1879 (2) Å
*b* = 19.9274 (7) Å
*c* = 6.9846 (2) Å
β = 91.199 (1)°
*V* = 861.07 (5) Å^3^
*Z* = 4
Mo *K*α radiation
μ = 0.11 mm^−1^
*T* = 293 K
0.30 × 0.25 × 0.20 mm

#### Data collection

Bruker SMART APEXII area-detector diffractometer
Absorption correction: multi-scan (*SADABS*; Bruker, 2008 ▶) *T*_min_ = 0.968, *T*_max_ = 0.979
12570 measured reflections
1763 independent reflections
1437 reflections with *I* > 2σ(*I*)
*R*_int_ = 0.024

#### Refinement

*R*[*F* ^2^ > 2σ(*F* ^2^)] = 0.037
*wR*(*F* ^2^) = 0.111
*S* = 1.04
1763 reflections
126 parameters
H atoms treated by a mixture of independent and constrained refinement
Δρ_max_ = 0.19 e Å^−3^
Δρ_min_ = −0.17 e Å^−3^

### 

Data collection: *APEX2* (Bruker, 2008 ▶); cell refinement: *SAINT* (Bruker, 2008 ▶); data reduction: *SAINT*; program(s) used to solve structure: *SHELXS97* (Sheldrick, 2008 ▶); program(s) used to refine structure: *SHELXL97* (Sheldrick, 2008 ▶); molecular graphics: *ORTEP-3 for Windows* (Farrugia, 2012 ▶) and *PLATON* (Spek, 2009 ▶); software used to prepare material for publication: *SHELXL97* and *PLATON* (Spek, 2009 ▶).

## Supplementary Material

Crystal structure: contains datablock(s) global, I. DOI: 10.1107/S1600536813015328/su2605sup1.cif

Structure factors: contains datablock(s) I. DOI: 10.1107/S1600536813015328/su2605Isup2.hkl

Supplementary material file. DOI: 10.1107/S1600536813015328/su2605Isup3.cml

Additional supplementary materials:  crystallographic information; 3D view; checkCIF report

## Figures and Tables

**Table 1:** Hydrogen-bond geometry (Å, °)

*D*—H⋯*A*	*D*—H	H⋯*A*	*D*⋯*A*	*D*—H⋯*A*
O3—H3*A*⋯N2^i^	0.82	1.82	2.6210 (16)	167
N2—H2*A*⋯O1	0.796 (19)	2.58 (2)	3.2437 (17)	141.4 (17)
N2—H2*A*⋯O2	0.796 (19)	2.557 (19)	3.2273 (17)	142.8 (19)
C2—H2⋯O1^i^	0.93	2.51	3.3428 (17)	149
C6—H6⋯O3^ii^	0.93	2.57	3.5035 (17)	179

Symmetry codes: (i) 
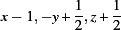
; (ii) 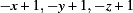.

**Fig. 1.**
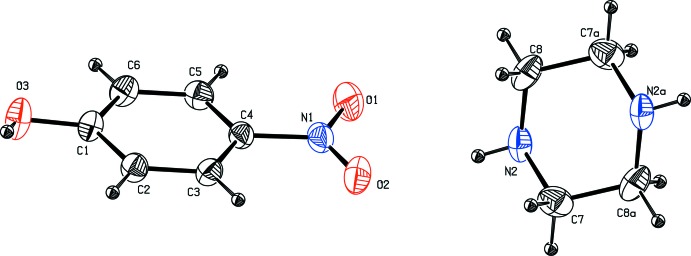
The molecular structure of the title compound, with atom labelling. Displacement ellipsoids are drawn at the 30% probability level (symmetry code: (a) = -x+2, -y, -z+1).

**Fig. 2.**
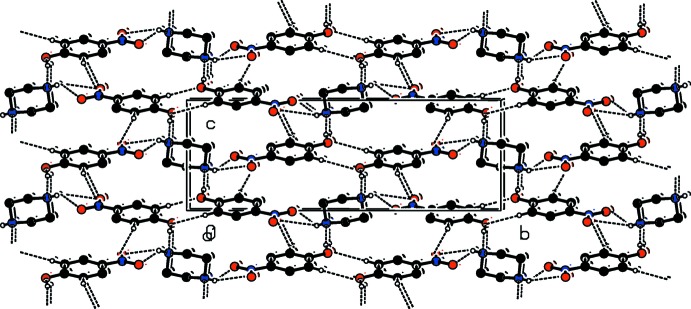
The crystal packing of the title compound viewed along the a axis. The N-H···O, O-H···N and C-H···O hydrogen bonds are shown as dashed lines; see Table 1 for details. The H atoms not involved in hydrogen bonding have been omitted for clarity.

## supplementary crystallographic information

### Comment

Piperazine-based research has attracted considerable attention in recent years. A broad range of compounds displaying antibacterial (Foroumadi *et al.*, 2007), antifungal (Upadhayaya *et al.*, 2004), anticancer (Chen *et al.*, 2006), antiparasitic (Cunico *et al.*, 2009), antihistamin (Smits *et al.*, 2008), and antidepressive activities (Becker *et al.*, 2006) have been found to contain this versatile core. In view of these important properties, we have undertaken the X-ray diffraction study of the title compound.

In the title adduct, C_6_H_5_N_1_O_3_, 0.5(C_4_H_10_N_2_), the piperazine ring (N2/C7/C8/N2a/C7a/C8a) possesses inversion symmetry. It adopts a *chair* conformation and its mean plane makes a dihedral angle of 65.45 (7)° with the 4-nitrophenol ring (C1-C6).

In the crystal, the piperazine ring is linked to two 4-nitrophenol molecules via O-H···N hydrogen bonds (Table 1 and Fig 2). The molecules are also linked via bifurcated N-H···O/O hydrogen bonds, involving the NO_2_ O atoms, forming a two-dimensional network lying parallel to (102). These networks are linked via C-H···O hydrogen bonds forming a three-dimensional structure (Table 1).

### Experimental

Piperazine 4-nitrophenol was synthesized by mixing an equimolar mixture (1:1) of anhydrous piperazine and 4-nitrophenol in methanol. The resultant solution was stirred magnetically at room temperature and filtered into a clean beaker. The filtrate was kept in a constant temperature bath at 308 K. Yellow block-like crystals suitable for x-ray diffraction were harvested from the solution within a day.

### Refinement

The OH and C-bound H atoms were positioned geometrically and refined using a riding model: O—H = 0.82 Å, C—H = 0.93 and 0.97 Å for aryl and methylene H-atoms, respectively, with U_iso_(H) = 1.5U_eq_(O) and = 1.2U_eq_(C,N).

### Crystal data

**Table:**

C_6_H_5_NO_3_·0.5C_4_H_10_N_2_	*F*(000) = 384
*M**_r_* = 182.18	*D*_x_ = 1.405 Mg m^−^^3^
Monoclinic, *P*2_1_/*c*	Mo *K*α radiation, λ = 0.71073 Å
Hall symbol: -P 2ybc	Cell parameters from 1763 reflections
*a* = 6.1879 (2) Å	θ = 2.0–26.4°
*b* = 19.9274 (7) Å	µ = 0.11 mm^−^^1^
*c* = 6.9846 (2) Å	*T* = 293 K
β = 91.199 (1)°	Block, yellow
*V* = 861.07 (5) Å^3^	0.30 × 0.25 × 0.20 mm
*Z* = 4	

### Data collection

**Table:**

Bruker SMART APEXII area-detector diffractometer	1763 independent reflections
Radiation source: fine-focus sealed tube	1437 reflections with *I* > 2σ(*I*)
Graphite monochromator	*R*_int_ = 0.024
ω and φ scans	θ_max_ = 26.4°, θ_min_ = 2.0°
Absorption correction: multi-scan (*SADABS*; Bruker, 2008)	*h* = −7→7
*T*_min_ = 0.968, *T*_max_ = 0.979	*k* = −24→24
12570 measured reflections	*l* = −8→8

### Refinement

**Table:**

Refinement on *F*^2^	Secondary atom site location: difference Fourier map
Least-squares matrix: full	Hydrogen site location: inferred from neighbouring sites
*R*[*F*^2^ > 2σ(*F*^2^)] = 0.037	H atoms treated by a mixture of independent and constrained refinement
*wR*(*F*^2^) = 0.111	*w* = 1/[σ^2^(*F*_o_^2^) + (0.0534*P*)^2^ + 0.1751*P*] where *P* = (*F*_o_^2^ + 2*F*_c_^2^)/3
*S* = 1.04	(Δ/σ)_max_ < 0.001
1763 reflections	Δρ_max_ = 0.19 e Å^−^^3^
126 parameters	Δρ_min_ = −0.17 e Å^−^^3^
0 restraints	Extinction correction: *SHELXL*, Fc^*^=kFc[1+0.001xFc^2^λ^3^/sin(2θ)]^-1/4^
Primary atom site location: structure-invariant direct methods	Extinction coefficient: 0.022 (4)

### Special details

**Table:**

Geometry. All esds (except the esd in the dihedral angle between two l.s. planes) are estimated using the full covariance matrix. The cell esds are taken into account individually in the estimation of esds in distances, angles and torsion angles; correlations between esds in cell parameters are only used when they are defined by crystal symmetry. An approximate (isotropic) treatment of cell esds is used for estimating esds involving l.s. planes.
Refinement. Refinement of F^2^ against ALL reflections. The weighted R-factor wR and goodness of fit S are based on F^2^, conventional R-factors R are based on F, with F set to zero for negative F^2^. The threshold expression of F^2^ > 2sigma(F^2^) is used only for calculating R-factors(gt) etc. and is not relevant to the choice of reflections for refinement. R-factors based on F^2^ are statistically about twice as large as those based on F, and R- factors based on ALL data will be even larger.

### Fractional atomic coordinates and isotropic or equivalent isotropic displacement parameters (Å^2^)

**Table:**

	*x*	*y*	*z*	*U*_iso_*/*U*_eq_	
C1	0.3284 (2)	0.39314 (6)	0.57841 (18)	0.0431 (3)	
C2	0.2308 (2)	0.33121 (6)	0.61501 (19)	0.0446 (3)	
H2	0.0929	0.3300	0.6654	0.053*	
C3	0.3365 (2)	0.27233 (6)	0.57715 (19)	0.0436 (3)	
H3	0.2711	0.2313	0.6019	0.052*	
C4	0.5409 (2)	0.27456 (6)	0.50202 (18)	0.0412 (3)	
C5	0.6389 (2)	0.33516 (7)	0.45997 (18)	0.0460 (3)	
H5	0.7756	0.3359	0.4072	0.055*	
C6	0.5330 (2)	0.39403 (7)	0.4966 (2)	0.0474 (3)	
H6	0.5974	0.4348	0.4670	0.057*	
C7	0.8041 (2)	−0.00321 (8)	0.3919 (2)	0.0604 (4)	
H7A	0.7274	−0.0085	0.2703	0.073*	
H7B	0.6982	0.0024	0.4911	0.073*	
C8	1.0620 (3)	0.06407 (7)	0.5674 (2)	0.0615 (4)	
H8A	0.9605	0.0707	0.6697	0.074*	
H8B	1.1545	0.1033	0.5618	0.074*	
N1	0.65536 (19)	0.21262 (6)	0.46944 (16)	0.0506 (3)	
N2	0.9439 (2)	0.05619 (6)	0.38520 (18)	0.0515 (3)	
O1	0.84102 (19)	0.21550 (6)	0.4113 (2)	0.0764 (4)	
O2	0.56486 (19)	0.15891 (5)	0.50020 (17)	0.0672 (3)	
O3	0.23383 (18)	0.45125 (5)	0.61778 (16)	0.0628 (3)	
H3A	0.1330	0.4447	0.6898	0.094*	
H2A	0.867 (3)	0.0873 (10)	0.362 (3)	0.071 (5)*	

### Atomic displacement parameters (Å^2^)

**Table:**

	*U*^11^	*U*^22^	*U*^33^	*U*^12^	*U*^13^	*U*^23^
C1	0.0508 (7)	0.0362 (6)	0.0425 (7)	0.0028 (5)	0.0080 (5)	−0.0010 (5)
C2	0.0394 (6)	0.0439 (7)	0.0507 (7)	0.0000 (5)	0.0092 (5)	−0.0019 (5)
C3	0.0464 (7)	0.0370 (6)	0.0473 (7)	−0.0033 (5)	0.0015 (6)	−0.0022 (5)
C4	0.0439 (7)	0.0420 (7)	0.0376 (6)	0.0076 (5)	−0.0014 (5)	−0.0052 (5)
C5	0.0397 (7)	0.0547 (8)	0.0438 (7)	0.0013 (5)	0.0079 (5)	−0.0017 (6)
C6	0.0521 (8)	0.0420 (7)	0.0486 (7)	−0.0058 (6)	0.0106 (6)	0.0011 (5)
C7	0.0445 (7)	0.0688 (10)	0.0680 (9)	−0.0099 (7)	0.0014 (6)	0.0101 (8)
C8	0.0785 (10)	0.0362 (7)	0.0703 (10)	−0.0139 (7)	0.0146 (8)	−0.0050 (6)
N1	0.0530 (7)	0.0529 (7)	0.0458 (6)	0.0152 (5)	−0.0031 (5)	−0.0079 (5)
N2	0.0537 (7)	0.0356 (6)	0.0658 (8)	0.0096 (5)	0.0141 (6)	0.0094 (5)
O1	0.0564 (7)	0.0762 (8)	0.0972 (10)	0.0210 (6)	0.0138 (6)	−0.0163 (6)
O2	0.0812 (8)	0.0428 (6)	0.0776 (8)	0.0125 (5)	0.0034 (6)	−0.0018 (5)
O3	0.0740 (7)	0.0385 (5)	0.0772 (8)	0.0080 (5)	0.0329 (6)	0.0014 (5)

### Geometric parameters (Å, º)

**Table:**

C1—O3	1.3290 (15)	C7—N2	1.4673 (18)
C1—C6	1.3995 (19)	C7—C8^i^	1.493 (2)
C1—C2	1.4000 (17)	C7—H7A	0.9700
C2—C3	1.3720 (18)	C7—H7B	0.9700
C2—H2	0.9300	C8—N2	1.463 (2)
C3—C4	1.3799 (19)	C8—C7^i^	1.493 (2)
C3—H3	0.9300	C8—H8A	0.9700
C4—C5	1.3855 (18)	C8—H8B	0.9700
C4—N1	1.4434 (16)	N1—O1	1.2279 (16)
C5—C6	1.3706 (18)	N1—O2	1.2290 (16)
C5—H5	0.9300	N2—H2A	0.80 (2)
C6—H6	0.9300	O3—H3A	0.8200
			
O3—C1—C6	118.65 (11)	N2—C7—H7A	109.7
O3—C1—C2	122.45 (12)	C8^i^—C7—H7A	109.7
C6—C1—C2	118.90 (11)	N2—C7—H7B	109.7
C3—C2—C1	120.60 (12)	C8^i^—C7—H7B	109.7
C3—C2—H2	119.7	H7A—C7—H7B	108.2
C1—C2—H2	119.7	N2—C8—C7^i^	110.09 (12)
C2—C3—C4	119.37 (12)	N2—C8—H8A	109.6
C2—C3—H3	120.3	C7^i^—C8—H8A	109.6
C4—C3—H3	120.3	N2—C8—H8B	109.6
C3—C4—C5	121.15 (11)	C7^i^—C8—H8B	109.6
C3—C4—N1	119.28 (12)	H8A—C8—H8B	108.2
C5—C4—N1	119.57 (12)	O1—N1—O2	122.10 (12)
C6—C5—C4	119.55 (12)	O1—N1—C4	118.54 (12)
C6—C5—H5	120.2	O2—N1—C4	119.36 (12)
C4—C5—H5	120.2	C8—N2—C7	110.05 (11)
C5—C6—C1	120.37 (12)	C8—N2—H2A	112.3 (14)
C5—C6—H6	119.8	C7—N2—H2A	106.6 (14)
C1—C6—H6	119.8	C1—O3—H3A	109.5
N2—C7—C8^i^	109.63 (11)		
			
O3—C1—C2—C3	−178.17 (12)	O3—C1—C6—C5	177.78 (12)
C6—C1—C2—C3	2.1 (2)	C2—C1—C6—C5	−2.5 (2)
C1—C2—C3—C4	−0.1 (2)	C3—C4—N1—O1	−176.52 (12)
C2—C3—C4—C5	−1.5 (2)	C5—C4—N1—O1	2.59 (19)
C2—C3—C4—N1	177.55 (11)	C3—C4—N1—O2	3.58 (19)
C3—C4—C5—C6	1.2 (2)	C5—C4—N1—O2	−177.31 (11)
N1—C4—C5—C6	−177.93 (11)	C7^i^—C8—N2—C7	59.04 (16)
C4—C5—C6—C1	0.9 (2)	C8^i^—C7—N2—C8	−58.77 (17)

Symmetry code: (i) −*x*+2, −*y*, −*z*+1.

### Hydrogen-bond geometry (Å, º)

**Table:**

*D*—H···*A*	*D*—H	H···*A*	*D*···*A*	*D*—H···*A*
O3—H3*A*···N2^ii^	0.82	1.82	2.6210 (16)	167
N2—H2*A*···O1	0.796 (19)	2.58 (2)	3.2437 (17)	141.4 (17)
N2—H2*A*···O2	0.796 (19)	2.557 (19)	3.2273 (17)	142.8 (19)
C2—H2···O1^ii^	0.93	2.51	3.3428 (17)	149
C6—H6···O3^iii^	0.93	2.57	3.5035 (17)	179

Symmetry codes: (ii) *x*−1, −*y*+1/2, *z*+1/2; (iii) −*x*+1, −*y*+1, −*z*+1.

## References

[bb1] Becker, O. M., Dhanoa, D. S., Marantz, Y., Chen, D., Shacham, S., Cheruku, S., Heifetz, A., Mohanty, P., Fichman, M., Sharadendu, A., Nudelman, R., Kauffman, M. & Noiman, S. (2006). *J. Med. Chem* **49**, 3116–3135. 10.1021/jm0508641 16722631

[bb2] Bruker (2008). *APEX2, *SAINT** and *SADABS* Bruker AXS Inc., Madison, Wisconsin, U. S. A.

[bb3] Chen, J. J., Lu, M., Jing, Y. K. & Dong, J. H. (2006). *Bioorg. Med. Chem* **14**, 6539–6547. 10.1016/j.bmc.2006.06.013 16806947

[bb4] Cunico, W., Gomes, C. R. B., Moreth, M., Manhanini, D. P., Figueiredo, I. H., Penido, C., Henriques, M. G. M. O., Varotti, F. P. & Krettli, A. U. (2009). *Eur. J. Med. Chem* **44**, 1363–1368. 10.1016/j.ejmech.2008.04.009 18514971

[bb5] Farrugia, L. J. (2012). *J. Appl. Cryst.* **45**, 849–854.

[bb6] Foroumadi, A., Emami, S., Mansouri, S., Javidnia, A., Saeid-Adeli, N., Shirazi, F. H. & Shafiee, A. (2007). *Eur. J. Med. Chem* **42**, 985–992. 10.1016/j.ejmech.2006.12.034 17316916

[bb7] Sheldrick, G. M. (2008). *Acta Cryst.* A**64**, 112–122. 10.1107/S0108767307043930 18156677

[bb8] Smits, R. A., Lim, H. D., Hanzer, A., Zuiderveld, O. P., Guaita, E., Adami, M., Coruzzi, G., Leurs, R. & Esch, I. J. P. (2008). *J. Med. Chem* **51**, 2457–2467. 10.1021/jm7014217 18357976

[bb9] Spek, A. L. (2009). *Acta Cryst.* D**65**, 148–155. 10.1107/S090744490804362X PMC2631630 19171970

[bb10] Upadhayaya, R. S., Sinha, N., Jain, S., Kishore, N., Chandra, R. & Arora, S. K. (2004). *Bioorg. Med. Chem* **12**, 2225–2238. 10.1016/j.bmc.2004.02.014 15080922

